# EGFR protein overexpression correlates with chromosome 7 polysomy and poor prognostic parameters in clear cell renal cell carcinoma

**DOI:** 10.1186/1423-0127-19-40

**Published:** 2012-04-05

**Authors:** Gordana Đorđević, Koviljka Matušan Ilijaš, Ita Hadžisejdić, Anton Maričić, Blaženka Grahovac, Nives Jonjić

**Affiliations:** 1 Department of Pathology, School of Medicine, University of Rijeka, B. Branchetta 20, Rijeka 51000, Croatia; 2 Department of Urology, Rijeka University Hospital Centre, Krešimirova 42, Rijeka 51000, Croatia

**Keywords:** Carcinoma, Renal cell, Chromosome 7, EGFR, In Situ Hybridization, Fluorescence, Prognosis

## Abstract

**Background:**

The role of epidermal growth factor (EGF) and its receptor (EGFR) in the pathogenesis and progression of various malignant tumors has long been known, but there is still disagreement concerning prognostic significance of EGFR expression in clear cell renal cell carcinoma (CCRCC). The present study was designed to analyze more objectively the protein EGFR expression in CCRCC and to compare its value with EGFR gene copy number changes and clinicopathologic characteristics including patient survival.

**Methods:**

The protein EGFR expression was analyzed immunohistochemically on 94 CCRCC, and gene copy number alterations of EGFR by FISH analysis on 41 CCRCC selected according to distinct membrane EGFR staining.

**Results:**

Membrane EGFR expression in tumor cells was heterogeneous with respect to the proportion of positive cells and staining intensity. FISH analysis did not reveal EGFR gene amplification, while polysomy of chromosome 7 found in 41% was associated with higher EGFR membrane expression. Moreover, EGFR overexpression was associated with a higher nuclear grade, larger tumor size and shorter patient's survival, while there was no connection with pathological stage.

**Conclusion:**

In conclusion, the protein expression of EGFR had an impact on prognosis in patients with CCRCC, while an increased copy number of chromosome 7 could be the possible reason for EGFR protein overexpression in the absence of gene amplification.

## Background

The role of growth factors in the pathogenesis and progression of various malignant tumors has long been known [1-3]. Among them, epidermal growth factor (EGF) and its receptor (EGFR) play a central role. Specific ligands, EGF and related growth factors such as TGF α, ampiregulin, betacellulin, neuregulins, epiregulin and heparin binding growth factor bind to the extracellular domain of EGFR resulting in receptor conformational change. This structural change allows for receptor dimerization and autophosphorylation of tyrosine kinase residues within the intracellular domain leading to activation of the signal transduction pathways. EGFR tyrosine phosphorylation triggers several signaling cascades, including the RAS-MAPK, PI3K-Akt and STAT pathways. Together, these EGFR-induced signaling pathways control gene transcription, cell cycle progression, cell proliferation and survival, adhesion, angiogenesis, migration, and invasion [4].

EGFR may be deregulated following point mutations occurring in the tyrosine kinase (TK) domain or protein overexpression. Both mechanisms can constitutively activate EGFR in a ligand independent manner [5,6]. Several reports indicate that an increased gene copy number of EGFR or mutations within the genes responsible for downstream signaling are important determinants of response or resistance to anti-EGFR antibodies [7]. Evaluation of EGFR by immunohistochemistry as a screening method on paraffin embedded tumor tissues has been widely used recently, primarily to select patients for targeted therapies. However, at practical level, immuno-determination of EGFR overexpression does not seem to accurately predict response to EGFR targeted therapies. It has been shown that only EGFR activating mutations or gene amplification seem to have a strong predictive value [8-10]. Although prognostic significance of EGFR was confirmed in numerous studies [11-13], the association between EGFR expression and prognosis in clear cell renal cell carcinoma (CCRCC) is still controversial [14-16]. Overexpression of EGFR in renal cell carcinoma (RCC) has been shown in various research, ranging from 40-80%. Evaluation of EGFR immunoexpression is not yet standardized and different scoring systems have been reported. Furthermore, positivity of staining in most studies has been defined only upon the membranous EGFR expression and cytoplasmic staining was not considered as positive. Pu at al suggests that different locations of EGFR expression may be associated with human renal tumorigenesis [14-16].

Thus, the aim of the present study was to analyze protein expression of EGFR by immunohistochemistry and to investigate the role of EGFR gene copy number changes in relation to EGFR overexpression in this type of renal cancer. Study parameters were compared with common clinicopathologic characteristics including patient survival. Thus, the aim of the present study was to analyze protein expression of EGFR by immunohistochemistry and to investigate the role of EGFR gene copy number changes in relation to EGFR overexpression in this type of renal cancer. Study parameters were compared with common clinicopathologic characteristics including patient survival.

## Methods

### Patients and tumor specimens

Tissue microarrays (TMA) were built from 94 archive formalin fixed and paraffin embedded CCRCC tissues from Department of Pathology, School of Medicine, University of Rijeka, Rijeka, Croatia, collected consecutively from 1989 to 1994. The representative areas on the haematoxylin and eosin (HE) stained sections were carefully selected and marked on corresponding paraffin blocks [17]. From each primary carcinoma, two tissue cores (1 mm in diameter) were obtained and arrayed in a recipient paraffin block using MTA Booster OI manual tissue arrayer (Alphelys, Plaisir, France).

Tumors were classified and staged according to the WHO [18] and TNM classification of renal cell neoplasm [19]. Grading of CCRCC was assessed according to the four-tiered system of Fuhrman *et al. *[20]. Clinicopathologic data including age, gender, tumor size, pathological T stage, nuclear grade and patient survival are summarized in Table 1.

**Table 1:** Clinicopathological parameters of CCRCC (N = 94)

Patients age (years), median (range)	61 (27-82)
Gender, No. (%)	
M	59 (62.8)
F	35 (37.2)
Tumor size (cm), median (range)	6.3 (1.8-17.5)
Fuhrman nuclear grade, No. (%)	
1	12 (12.8)
2	40 (42.6)
3	22 (23.4)
4	20 (21.2)
Pathological T stage (pT), No. (%)	
1	49 (52.1)
2	22 (23.4)
3	23 (24.5)
4	0 (0)
Patient survival (months), median (range)	64 (1-165)

CCRCC, clear cell renal cell carcinoma; M, male; F, female

### Immunohistochemistry and evaluation

Immunohistochemistry was performed on 4-μm thick paraffin sections using EGFR mouse monoclonal antibody (IgG_1 _clone 2-18 C9; pharm Dx™ ready to use kit Dako Cytomation, Glostrup, Denmark) as primary antibody. According to the manufacturer's instructions, for EGFR staining we employed antigen retrieval using proteinase K. Standard immunohistochemistry procedure was performed in Dako Autostainer Plus (DakoCytomation Colorado Inc, Fort Collins, CO, USA) according to the manufacturer's instructions, using appropriate DakoREAL solutions (Dako, Glostrup, Denmark). Breast cancer tissues served as a positive control of EGFR. Also, a negative control consisting of the omission of the primary antibody was performed for each case.

**Figure 1:**
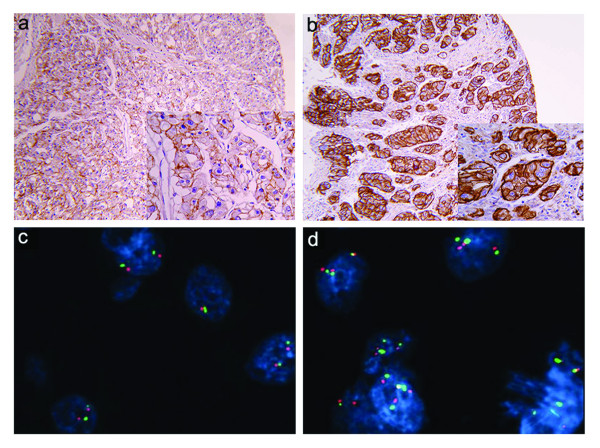
**EGFR protein and gene status in clear cell renal cell carcinoma**. Immunohistochemical expression of EGFR protein, shown at lower (×10) and higher magnification (×20), was designated as discontinuous or continuous membrane staining of different intensity: (**a) **weak and moderate (+/++) discontinuous and continuous membrane immunostaining, and (**b**) strong continuous immunostaining (+++);. (**c**) chromosome 7 copy number was analyzed in tumor cells using fluorescence in situ hybridization (FISH) with an α-satellite DNA probe for chromosome 7 (centromere 7, green signal; EGFR gene, red signal); tumor nuclei showed disomy of chromosome 7 without EGFR gene amplification (**d**) tumor nuclei showed polysomy with a greater number of red and green signals than in normal cells; (**c **and **d **magnification × 100).

EGFR staining patterns were designated as cytoplasmic or membranous. The membranous pattern was further classified as partial (discontinuous) (Figure 1a) or circumferential (continuous) (Figure 1b) membrane positivity, while staining intensity was graded as 0 for negative, 1+ for weak, 2+ for moderate, and 3+ for strong staining. Evaluation of immunohistochemical staining was based on histo score (H-score) that was calculated by multiplying the intensity of EGFR staining with percentage of tumor cells showing particular membranous staining pattern [15]. Continuous H-score (cH-score) was considered for only continuous (circumferential) staining of cell membrane, while total H-score (tH-score) included any membrane staining (continuous and discontinuous). For the purpose of two-way statistical analysis, H-score was determined as "low" or "high" using the median as the cut-off value. The evaluation of immunostaining was performed by one pathologist (G. Đ.), who was blinded for clinical data. On statistical analysis, the mean value of immunohistochemical staining of two tissue microarrays was used.

### EGFR fluorescent in situ hybridization (FISH)

Forty-one tumors with distinct membrane EGFR immunoexpression on at least 5% of tumor cells were selected for FISH analysis, or more precisely, 14 tumors with strong (3+) and 16 with moderate (2+) continuous membrane staining, and 11 tumors with week (1+) continuous and discontinuous membrane staining. Also, FISH analysis was performed on the rest of the CCRCC sample in order to assess EGFR gene copy number changes in all tumors regardless of the EGFR membrane immunohistochemical pattern.

Three-μm thick sections were cut from TMA paraffin blocks and used for FISH analysis. For proteolytic TMA slide pretreatment, a commercial kit was used (Paraffin pretreatment reagent kit, Vysis, Downers Grove, USA) according to the manufacturer's instructions. We used LSI EGFR SpectrumOrange/CEP 7 SpectrumGreen probe (Abbott, Vysis, Downers Grove, IL, USA), which hybridizes to the EGFR gene (orange signal) and to the centromeric region of chromosome 7 (green signal). Briefly, TMA sections were deparaffinized in xylene substitute, rinsed in 100% ethanol and air dried. Subsequently, slides were incubated in 0.2 N HCl for 20 min, rinsed in 2xSSC, pH 7.0 and immersed for 30 min in 1 M NaSCN solution pre-warmed at 80°C. After proteinse digestion, slide denaturation (95°C for 5 min) and hybridization (37°C overnight) were carried out in HYBrite™ (Vysis, IL, USA). On the next day, the slides were washed, counterstained with DAPI (Vysis, Downers Grove, IL, USA) and examined under fluorescent microscope (Olympus BX50, Tokyo, Japan).

At least 20 nuclei were analyzed from each tumor at three representative tumor areas *per *two tissue cores. Cells were selected for scoring according to morphological criteria, where only those nuclei having an evident malignant cytological appearance were scored. Small cells, overlapping or damaged nuclei were disregarded. A cell with a normal number of copies of the EGFR gene or chromosome 7 status was characterized by 2 orange and 2 green signals *per *nucleus. A tumor was characterized as polysomic if there were more than two centromere 7 signals *per *cell (polysomy) in more than 20% of tumor cells [21]. EGFR amplification was defined as the presence of the oncogene/centromere ratio ≥ 2.0.

### Statistical analysis

Statistical analysis was performed using Statistica software (StatSoft, Inc., Tulsa, OK, USA). The distribution of data was tested for normality using Kolmogorov-Smirnov test. Tumor size and chromosome 7 polysomy were used as numerical data, while nuclear grade, pathological T stage, EGFR H-score and chromosome 7 polysomy as categorical data were divided in two categories. Pearson's χ^2^-test was used to assess the association of EGFR H-score with nuclear grade and pathological T stage, as well as to assess the association of EGFR staining intensity and chromosome 7 polysomy status in CCRCC. Association between EGFR protein expression and tumor size was defined with Mann-Whitney U-test. One -way ANOVA served to compare the EGFR immunohistochemical staining intensity and number of tumor cells showing chromosome 7 polysomy. Association between EGFR protein expression and overall patient survival was estimated using Kaplan-Meier method, while difference between survival rates was evaluated by use of log-rank test. Multivariate analysis was performed using Cox regression model. Statistical differences with a p value less than 0.05 were considered significant.

## Results

### Immunohistochemical and FISH analysis of EGFR expression

EGFR expression was largely undetected immunohistochemically in normal renal tissue with occasional weak basal cytoplasmic staining in proximal and distal tubules but not in collecting ducts or blood vessels. In tumor cells, cytoplasmic EGFR expression was very heterogeneous showing a small proportion of positive cells with weak intensity. More precisely, the cytoplasmic EGFR expression was found in 29/94 (30.8%) CCRCC tumors but ranged from 2% to 10% of positive tumor cells; therefore, it was not further commented.

Membrane EGFR expression in tumor cells was also heterogeneously distributed in respect to the proportion of positive tumor cells and staining intensity. The median percentage of membranous staining was 28.9%, ranging from 0% to 100%. When the percentage of membrane EGFR expression was combined with the intensity of staining, the median value of tH-score and cH-score was 30 (0-257) and 15.6 (0-255), respectively. A representative case with membranous EGFR expression in CCRCC is shown in Figure 1a and 1b.

**Figure 2:**
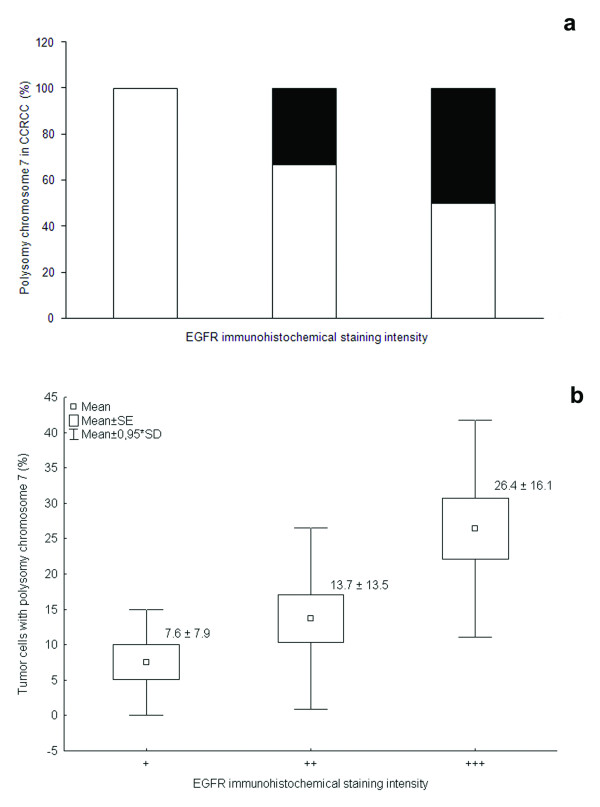
**Chromosome 7 polysomy in different groups of clear cell renal cell carcinoma (CCRCC) regarding EGFR immunohistochemical staining intensity:** (**a**) the number of tumors categorised as polysomic decreases with attenuation of staining intensity (p = 0.019, Pearson's χ^2^-test). Black bars represent percentage of tumors with chromosome 7 polysomy, white bars represent percentage of tumors without chromosome 7 polysomy; (**b**) the number of tumor cells showing chromosome 7 polysomy is declining with attenuation of EGFR immunohistochemical staining (p = 0.004, one-way ANOVA).

**Figure 3:**
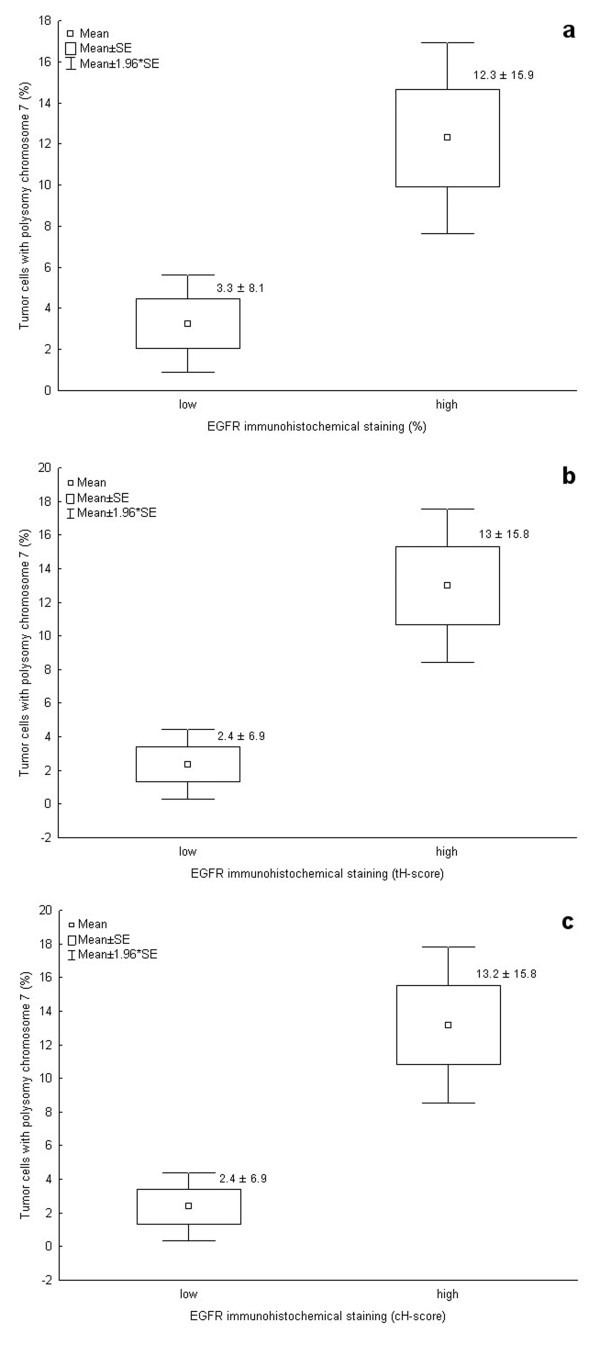
**Chromosome 7 polysomy and membrane EGFR protein expression determined as percentage of EGFR immunohistochemicaly positive cells per tumor (%), EGFR total H-score (tH-score) and EGFR continuous H-score (cH-score) in clear cell renal cell carcinoma (CCRCC)**. Low and high categories of EGFR immunohistochemical staining are designated according to median cut off value. Student's t-test is showing higher percentage of tumor cells with chromosome 7 polysomy to be significantly associated with high expression of all EGFR immunohistochemical staining categories: (a) EGFR % (p = 0.001); (b) EGFR tH-score (p < 0.001); (c) EGFR cH-score (p < 0.001).

FISH analysis did not reveal any amplification of EGFR gene in our cohort of CCRCC, while polysomy was detected in 17 of 41 examined tumors (41.5%) (Figure 1c and 1d). Chromosome 7 polysomy was found to be associated with EGFR immunohistochemical expression (Figure 2 and 3). The proportion of tumors showing chromosome 7 polysomy was increasing with EGFR staining intensity (p = 0.019) (Figure 2a). The EGFR staining intensity was also related to the number of tumor cells with chromosome 7 polysomy (p = 0.004) (Figure 2b).

In addition, comparison between EGFR FISH analysis and protein expression (determined as percentage of positive tumor cells, tH-score and cH-score) in CCRCC was assessed (Figure 3). Statistical analysis showed significant association between all EGFR immunohistochemical staining categories and percentage of positive tumor cells with chromosome 7 polysomy (Figure 3).

### Comparison of EGFR protein and gene status with clinicopathologic features

**Figure 4:**
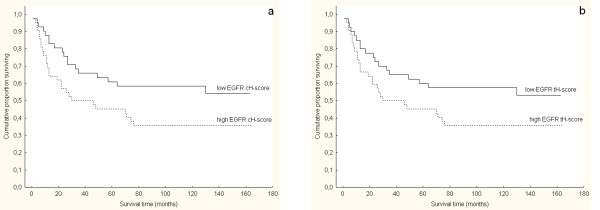
**Kaplan-Meier survival analysis according to EGFR protein expression determined as H-score of continuous immunohistochemical staining (cH-score) and total immunohistochemical staining (tH-score) in clear cell renal cell carcinoma (CCRCC):** (a) The log-rank test showed significantly shorter overall survival in patients with tumors showing high continuous EGFR protein expression that was above median H-score values (p = 0.046). The 5-year survival rate was 59% for patients whose tumors showed low EGFR cH-score *vs*. 40% for patients whose tumors showed high EGFR cH-score; (b) on the contrary, the log-rank test showed no significant association between EGFR tH-score and survival (p = 0.074). The 5-year survival rate was 57% for patients whose tumors showed low EGFR tH-score and 40% for patients whose tumors showed high EGFR tH-score.

EGFR immunoexpression analysis showed association of high %, tH-score and cH- score values with a higher nuclear grade (p < 0.001, p < 0.001 and p = 0.006, respectively) and larger tumors (p = 0.011, p = 0.002 and p = 0.01, respectively), while there was no association with pathological T stage (Table 2). On contrary, chromosome 7 polysomy showed no association with any clinicopathological feature as well as patients' survival. EGFR protein expression showed relationship with patient survival (Figure 4). Log-rank test showed an association between shorter patient survival and high EGFR protein expression only for continuous membranous EGFR staining (p = 0.046), while it could not be found for total membranous staining (p = 0.074) and % of EGFR membranous staining (p = 0.168). Association of EGFR cH-score and tH-score with cumulative proportion of patients surviving during the follow-up are shown in Figure 4. Univariate survival analysis also showed nuclear grade and pathological T stage to be significant predictive factors (p < 0.001 and p = 0.003, respectively). However, on multivariate analysis, only nuclear grade remained significant (p = 0.002, relative risk 3, 95% confidence interval 1.7-5.3), while pathologic stage (p = 0.009, relative risk 1.5, 95% confidence interval 1-2.4) together with EGFR protein expression (p = 0.175, relative risk 1.3, 95% confidence interval 0.9-1.9) showed no independent prognostic value.

**Table 2:** EGFR immunoexpression in relation to pathohistologic features of CCRCC (N = 94)

			**Nuclear grade**, N (%)		p value	**Tumor size**, median (range)	p value	**Pathological T stage**, N (%)		p value
			1, 2	3, 4				1, 2	3, 4	
EGFR immunoexpression ^a^,N (%)	%	low	33 (36.7)	12 (13.3)	< 0.001^b^	5 (1.8-17.5)	0.011^c^	32 (35.6)	13 (14.4)	0.213^b^
		high	16 (17.8)	29 (32.2)		7 (3-16)		37 (41.1)	8 (8.9)	
	tH-score	low	32 (35.9)	11 (12.4)	< 0.001^b^	5 (1.8-17.5)	0.002^c^	31 (34.8)	12 (13.5)	0.354^b^
		high	17 (19.1)	29 (32.6)		7 (3-16)		37 (41.6)	9 (10.1)	
	cH-score	low	31 (34.4)	14 (15.6)	0.006^b^	5 (1.8-17.5)	0.01^c^	33 (36.7)	12 (13.3)	0.454^b^
		high	18 (20)	27 (30)		7 (2.5-16)		36 (40)	9 (10)	

CCRCC, clear cell renal cell carcinoma

tH-score, total H-score

cH-score, continuous H-score

^a ^low and high categories according to median cut off value

^b ^chi square test

^c ^Mann-Whitney U-test

## Discussion

Many studies were conducted in order to determine whether EGFR protein overexpression or EGFR gene amplification could select patients who would benefit from anti EGFR therapy. Some clinical data do not support an association between EGFR immunohistochemical expression and response to specific targeted therapies [14]. On the contrary, a recent review by Martin *et al. *underlines that EGFR gene copy number changes detected by FISH analysis might be used as an effective tool in the selection of patient treatment [4]. In addition, many authors have shown that the immunohistochemistry based EGFR screening method is not strictly quantitative and needs standardized criteria for evaluation [8,22,23]. Thus, our study was undertaken and based on more objective evaluation of EGFR expression with the emphasis on comparison of protein expression value with FISH analysis.

Results of this study suggested two conclusions: first, that patients with strong EGFR membranous staining on at least 10% of tumor cells or with strong complete membrane staining on at least 5% of tumor cells have poorer prognosis; and second, that EGFR overexpression is not associated with gene amplification but most likely with polysomy of chromosome 7. From the practical point of view, we presume that these patients could possibly be selected for anti-EGFR therapy.

According to some studies, immunohistochemical expression of EGFR in RCC varies from 50% to 90% of tumor cells [24-26]. In our study, the mean percentage of positive cells, tH-score and cH-score was 29%, 30%, and 16%, respectively. Our results are somewhat lower when compared with H-score of 79 reported by Kallio *et al. *[25], but it should be noted that our study included only clear cell type of RCC, whereas the above mentioned authors focused on a heterogeneous group of tumor types. They also pinpointed papillary carcinomas to have the highest mean value, 140, of EGFR H-score, in comparison with other tumor types. This observation points to the importance of taking into account tumor heterogeneity when interpreting EGFR expression. The variability of findings with respect to EGFR expression in RCC could be explained by different histological types of study tumors and different methodology of evaluation used [23]. Langner *et al. *investigated whether heterogeneous results of prognostic significance of EGFR immunohistochemical findings in RCC were related to non-standardized criteria for staining evaluation [8]. This group of researchers took into consideration cytoplasmic EGFR immunostaining, which was associated with poor prognosis. We also examined cytoplasmic EGFR expression but it was not taken into consideration for further analysis because it was mainly weakly expressed in a low percentage of tumor cells (ranging from 2% to 10%).

The significance of continuous membrane HER-2 staining has been well recognized and included in pathological evaluation of breast cancer [27]. On the other hand, to our knowledge, we could not find any study on RCC where only complete membrane H-score of EGFR expression was identified and compared with clinicopathologic data. According to the results obtained in our study, the EGFR cH-scoring system should be taken into account because of its prognostic value, which is also supported by chromosomal abnormalities found by FISH analysis. Further investigations may shed more light on its therapeutic significance.

Studies investigating the prognostic value of EGFR expression in RCC show controversial results. In most studies, the high EGFR expression was associated with worse clinicopathologic prognostic parameters [28,29], as it was observed in the head and neck tumors, breast, lung and bladder cancer [4,30]. On the other hand, the minority of studies showed opposite results, or do not confirm the significant prognostic role of EGFR overexpression [15,16,25]. In relation to the clinical and pathological parameters, our univariate analysis confirmed both high tH-score and high cH-score to be associated with higher nuclear grade and larger tumor size. In contrast to our results, Kallio *et al. *found an association between higher EGFR expression and lower nuclear grade [25]. However, neither this nor our study found any connection between EGFR expression and tumor stage. The prognostic significance of EGFR expression was also confirmed in our survival analysis, however, only when the percentage of positive cells was estimated in association with staining intensity. Namely, the overall survival of patients with predominantly high EGFR cH-score expression was shorter. These results are in agreement with others studies in which strong membrane EGFR expression was significantly associated with worse survival [8,29,31].

The most common gene alteration that results in excessive expression of EGFR is amplification. Nonetheless, our FISH analysis confirmed that tumors with low or moderate EGFR membrane expression had no particular changes in the number of chromosome copies, and that those with strong EGFR expression had no gene amplification, as observed by some other authors [21,32]. On the other hand, chromosome 7 polysomy found by FISH analysis in our study was associated with strong and mostly continuous membrane expression, as also reported elsewhere [33].

## Conclusion

In conclusion, the EGFR protein expression has an impact on prognosis, i.e. a higher expression correlates with a higher nuclear grade and larger tumors, and strong continuous membrane immunostaining is significantly associated with shorter survival of patients with CCRCC. Increased copy numbers of chromosome 7 could be the source of overall EGFR expression at the protein level.

## Competing interests

The authors declare that they have no competing interests.

## Authors' contributions

GĐ conception and design, analysis and interpretation of data, drafting of the manuscript; KMI statistical analysis and data interpretaion; IH FISH analysis and data interpretation; AM provided clinical data and analyzed data; BG data analysis, critical evaluation of the manustcript draft; NJ conception and design, data interpretation; all authors read and approved the final manuscript.
